# TCR repertoires are skewed in South African patients with multisystem inflammatory syndrome in children

**DOI:** 10.1097/INF.0000000000004926

**Published:** 2025-08-06

**Authors:** Timothy F Spracklen, Mthawelanga Ndengane, Claire Butters, Myrsini Kaforou, Liesl J Zühlke, Michael Levin, Kate Webb

**Affiliations:** 1 Department of Paediatrics and Child Health, Faculty of Health Sciences, University of Cape Town, Cape Town, South Africa; 2 Cape Heart Institute, Faculty of Health Sciences, University of Cape Town, Cape Town, South Africa; 3 Department of Infectious Disease, Faculty of Medicine, Imperial College London, London, United Kingdom; 4 Centre for Paediatrics and Child Health, Imperial College London, London, United Kingdom; 5 South African Medical Research Council, Cape Town, South Africa; 6 Crick African Network, Francis Crick Institute, London, United Kingdom

**Keywords:** MIS-C, SARS-CoV-2, TRBV11-2

## Abstract

Multisystem inflammatory syndrome in children (MIS-C) is a severe response to SARS-CoV-2 that affects a small proportion of children. Overexpression of a nonspecific T cell receptor beta variable 11-2 (*TRBV11-2*) in multiple populations has led to the hypothesis of a superantigen response underlying MIS-C. Here, we describe *TRBV11-2* expansion in South African children with MIS-C, supporting a common pathogenesis of MIS-C regardless of location.

One of the early defining characteristics of the coronavirus-2019 (COVID-19) pandemic was that children were less severely affected by SARS-CoV-2 infection than adults. However, the emergence of multisystem inflammatory syndrome in children (MIS-C) in April 2020 showed that a small proportion of children are at risk of more severe responses to SARS-CoV-2 exposure. MIS-C is a post-infectious hyperinflammatory multiorgan condition which typically presents as a persistent fever with nonspecific symptoms such as rash, conjunctivitis, cardiac dysfunction and gastrointestinal problems. Despite the many clinical similarities with Kawasaki disease (KD) and toxic shock syndrome, immunopathological and laboratory profiling have characterized MIS-C as a distinct clinical entity.

It has been hypothesized that a superantigen response underlies MIS-C pathogenesis, triggering a cytokine storm that can cause tissue damage and autoantigen release. In support of this hypothesis, T cell receptor (TCR) Vβ skewing has been identified in MIS-C patients, in which expression of TCR Vβ21.3 (encoded by the gene *TRBV11-2*) was increased compared to controls, and associated with disease severity.^1^ *TRBV11-2* expansion has been reported in seven other studies from Europe, Japan and the United States, totaling over 170 MIS-C patients and a variety of controls, from healthy children to other febrile conditions including pediatric COVID-19, toxic shock syndrome and KD.^2–9^ It has been shown consistently that *TRBV11-2* expansion is unique to MIS-C and that this expansion is transient, returning to baseline shortly after the acute phase of MIS-C. However, it has been recently reported that expanded *TRBV11-2*+ T cells in MIS-C could be specific to Epstein-Barr virus (EBV), suggesting that the hyperinflammation is due to EBV reactivation and not a superantigen response.^10^

Due to its nonspecific symptoms, MIS-C can be difficult to distinguish from other pediatric infectious and inflammatory diseases, and these findings demonstrate the potential of *TRBV11-2* as a diagnostic biomarker MIS-C and/or for its severity. Indeed, *TRBV11-2* was described as part of a five-gene diagnostic transcriptomic signature that could distinguish MIS-C from other infectious diseases and KD, with *TRBV11-2* the most significantly different gene in the analysis.^11^

There have been exceedingly few reports of MIS-C in Africa, especially given the number of children who live on this vast continent.^12^ This may give an impression of rarity, although in areas with the resources to diagnose and record these cases, there was an estimated incidence of 22/100000 children, which matched incidences from better resourced settings,^13^ implying that the lack of reports did not reflect a lack of cases. Due to this disparity, it is important to investigate whether MIS-C had a similar pathogenic mechanism in African children in order to dispel the false impression that MIS-C did not affect African children. TCR repertoires in MIS-C have not been described in children outside of Europe, UK or the USA.

We have previously demonstrated the unique insights that can be gleaned when studying MIS-C in South African patients,^14^ with the implication that results from other settings may not always translate to Africa. We therefore aimed to characterize the TCR Vβ (TRBV) repertoires of MIS-C patients and controls from South Africa for whom RNA sequencing (RNAseq) data was available from the DIAMONDS study. The cohort consisted of 10 pre-treatment MIS-C patients, 10 healthy children and 16 contemporaneous febrile controls, as well as 8 matched post-treatment MIS-C specimens. The cohort was age- and sex-matched with no significant differences in these parameters between any of the groups (Supplemental Digital Content 1).

After normalization and quality control, differentially expressed TRBV genes were identified in the RNAseq dataset using DESeq2, in which the Holm-Bonferroni method was used to correct for multiple testing. TRBV usage was calculated as the number of read counts that mapped to each TRBV, divided by the total read counts that mapped to all TRBVs.

Our analysis identified 41 TRBVs in the dataset, with *TRBV11-2* the only gene that was significantly overexpressed in MIS-C compared to both controls (Figure 1A). *TRBV24-1* was only detected in healthy children and was absent in both MIS-C and febrile controls. We also observed a reduction in TRBV repertoire in MIS-C patients compared to healthy controls, suggestive of TCR skewing (Figure 1B). A similar reduction was seen in the febrile controls. While not statistically significant, *TRBV11-2* expression appeared to decrease and TCR diversity appeared to increase after the acute phase of disease, a median of 3 days after initiation of treatment. This is in accordance with other reports, which attribute *TRBV11-2* normalization to glucocorticoid use.^7, 9^ It should be noted that only three of the eight patients with follow-up data were treated with glucocorticoids in addition to intravenous immunoglobulin, and patients were followed up for shorter than the average 7 days in other reports. This may explain why *TRBV11-2* expression remained higher in MIS-C after treatment than in healthy controls and other febrile conditions (Figure 1A).

Five of the 10 MIS-C patients (50%) included in this analysis had severe disease requiring admission to an intensive care unit (ICU). While *TRBV11-2* usage did appear higher in this group compared to the five non-ICU patients (median: 0.463 vs 0.204), this was not statistically significant (Wilcoxon signed-rank test, p = 0.11), presumably due to the small numbers of observations in each group.

*TRBV11-2* usage correlated with C-reactive protein (CRP) levels in our patients (Figure 1C). It was also found to be a highly sensitive and specific biomarker for distinguishing MIS-C from febrile controls (Figure 1D).

This study was limited by the small numbers of participants in each group, and we were unfortunately unable to perform a sufficiently powered analysis of *TRBV11-2* usage by severity of disease as in previous studies. Nevertheless, we leveraged existing RNAseq data to independently validate the shift in TRBV repertoire with transient *TRBV11-2* expansion that has been observed in MIS-C in other settings. We demonstrate the high sensitivity and specificity of *TRBV11-2* expression as a potential diagnostic biomarker for acute MIS-C in African patients.

Future work will include validation of these findings in a larger cohort, as well as interrogation of African RNAseq data for additional differentially expressed genes and their overlaps with genetic signatures of MIS-C in other published populations. Further investigation is needed to determine whether the activation of *TRBV11-2*-expressing T cells underlies the development of MIS-C or is rather one of the mechanisms whereby autoimmune inflammatory responses are exacerbated in susceptible children.

## Supplementary Material

Supplementary table 1

## Figures and Tables

**Figure 1:**
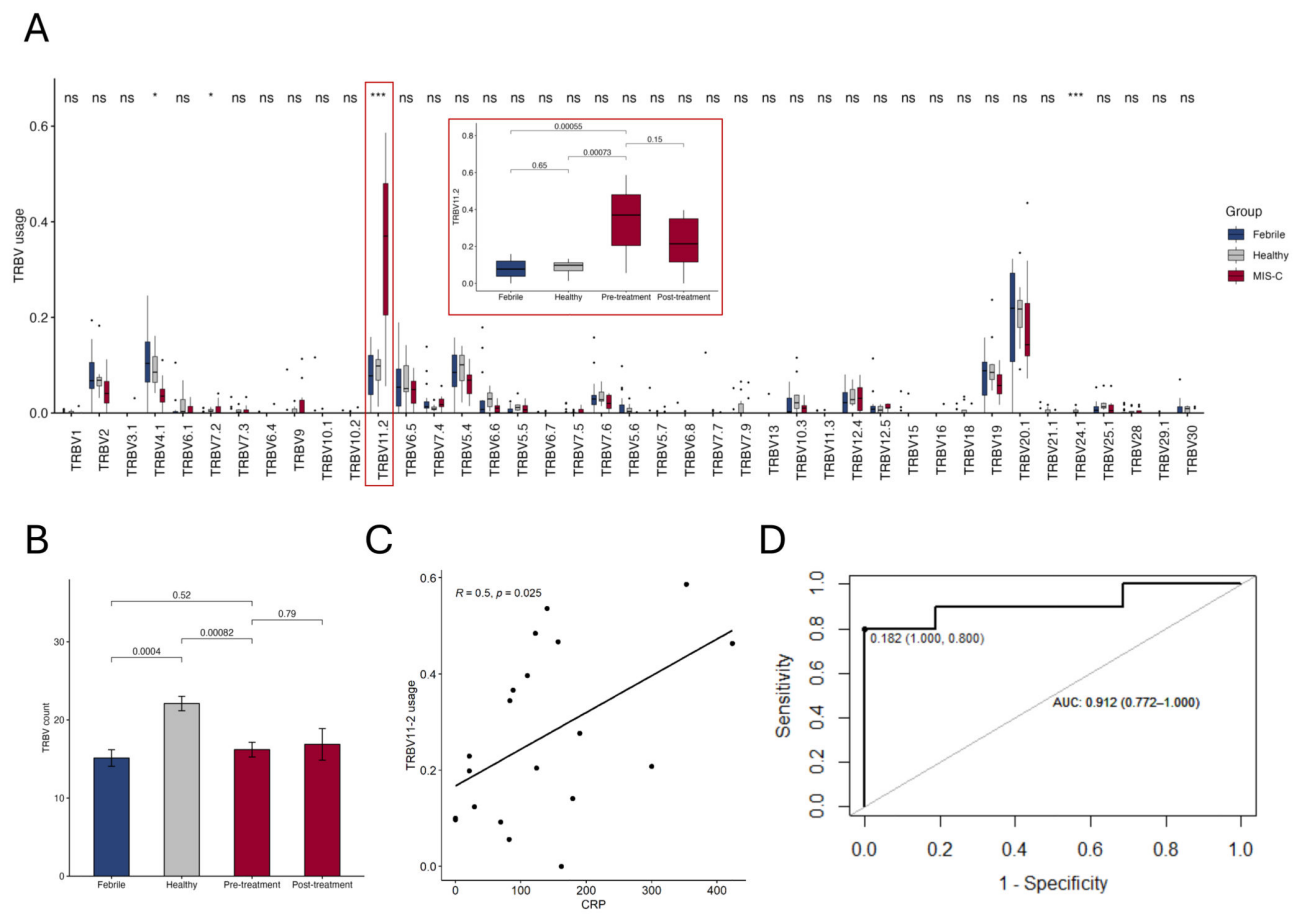
TCR profiling reveals skewed TRBV repertoires in South African MIS-C patients. (A) TRBV usage in MIS-C compared to healthy children and febrile controls. Inset, *TRBV11-2* usage in the controls and MIS-C before and after treatment. (B) TRBV diversity in healthy children and febrile controls compared to MIS-C before and after treatment. (C) Association of *TRBV11-2* usage and CRP levels in MIS-C patients. (D) Receiver operating curve analysis of the capacity of *TRBV11-2* expression to distinguish between MIS-C and febrile controls.
