# Supplementary material for: TCR repertoires are skewed in South African patients with multisystem inflammatory syndrome in children
Source: Pediatr Infect Dis J. Author manuscript; Available in PMC 2025 Dec 1. (PMC7618101; doi:10.1097/INF.0000000000004926)
Supplement: Supplementary table 1 [file EMS208395-supplement-Supplementary_table_1.pdf]

Supplementary table 1

Supplementary table 1: Clinical and demographic features of the RNAseq cohort

**Characteristic**	**MIS-C (n = 10)**	**Febrile controls (n = 16)**	**Healthy children (n = 10)**	**p-value**
Mean age (years)	7.5	6.4	6.8	0.750151[a]
Sex				
• Male	6 (60%)	11 (68.75%)	7 (70%)	0.896355[b]
• Female	4 (40%)	4 (25%)	3 (30%)	
• NR	-	1 (6.25%)	-	
Ethnicity				
• Black African	7 (70%)	6 (37.5%)	1 (10%)	**0.020355**[b]
• Admixed	3 (30%)	10 (62.5%)	9 (90%)	
Disease severity				
• Non-ICU	5 (50%)	15 (93.75%)	-	**0.018425**[b]
• ICU	5 (50%)	1 (6.25%)	-	
Median CRP (mg/L)	168	159	-	0.307720[c]
Diagnosis				
• MIS-C	10 (100%)	-	-	ND
• Definite/probable bacterial infection	-	10 (62.5%)	-	
• Definite/probable viral infection	-	2 (12.5%)	-	
• Inflammatory	-	4 (25%)	-	

CRP, C-reactive protein; ND, not done; NR, not recorded
[a] ANOVA; [b] Fisher exact test; [c] Mann Whitney U test
